# Physical activity in Germany: Discrepancy between high societal expectation and actual activity levels in old age

**DOI:** 10.25646/13553

**Published:** 2025-11-26

**Authors:** Nils Henrik Pixa, Eva-Marie Kessler, Lisa Marie Warner

**Affiliations:** 1 MSB Medical School Berlin, Department of Psychology, Berlin, Germany; 2 University of Münster, Institute of Sport and Exercise Sciences, Department of Neuromotor Behavior and Exercise, Münster, Germany

**Keywords:** Physical activity, Age, Prescriptive norm, Norm-behavior gap, Survey, Age_ISM Germany, GEDA, EHIS, Germany

## Abstract

**Background:**

Remaining physically active in later life has been shown to contribute to a longer and healthier life without the need for care or support. Accordingly, a societal expectation to stay active in old age (prescriptive age norm) has emerged – particularly among older adults themselves. Yet, are they in fact as physically active as expected?

**Methods:**

Using representative data from the Age_ISM Germany survey (ages 16 – 96 years), we examine the extent to which older individuals agree with the prescriptive age norm that ‘old people should normally remain physically active’ compared to younger individuals. This is contrasted with the actual activity behavior of 18- to 90-year-olds based on the 2019/2020 GEDA survey data from the Robert Koch Institute. This allows us to compare endorsement of the prescriptive norm with actual behavior.

**Results:**

Although the prescriptive age norm of remaining physically active is more strongly endorsed in the older age groups than in the younger age groups, the proportion of individuals who are sufficiently active according to the World Health Organization’s definition is significantly lower in the older age groups.

**Conclusions:**

The findings point to a ‘norm-behavior gap’: from around the age of 75 onwards, expectations regarding physical activity and actual behavior diverge increasingly. At the individual level, the negative age stereotype of older people as inactive and passive may, for instance, contribute to older adults’ perception of being less addressed by exercise programs. At the socio-structural level, this may indicate a lack of age-sensitive exercise programs.

## 1. Introduction

Physical activity has been shown to contribute to a healthier, more self-determined, and longer life with a higher quality of life. In contrast, insufficient physical activity increases the risk of chronic conditions such as type 2 diabetes, cardiovascular disease, obesity, and depression, while also having substantial health economic consequences [1, 2, 3]. In Germany, the annual excess costs for inactive individuals, compared to those who are sufficiently active, amount to around € 188 per person in direct healthcare expenditures and approximately € 482 per person when indirect costs are included [4]. In light of demographic change, these additional costs are expected to increase further, as older age groups – particularly those aged 60 years and over – are, on average, considerably less physically active than younger groups [1, 3, 4]. Taken together, these findings underline the need for effective public health strategies to promote physical activity across all age groups.

Since the 1990s, the World Health Organization (WHO) has pursued an *active aging strategy* and, since 2002, has explicitly recommended remaining physically active even in old age [5]. In Germany, the *National Recommendations for Physical Activity and Physical Activity Promotion* [6, 7] and the WHO’s physical activity guidelines [8, 9] currently serve as benchmarks for the extent, intensity, and type of health-enhancing physical activity for older adults [10]. The German National Physical Activity Recommendations are largely consistent with those issued by the WHO in 2010. These recommend engaging in at least 150 minutes per week of moderate-intensity, endurance-oriented aerobic physical activity (e.g. cycling, swimming, aerobics, rowing), or at least 75 minutes of vigorous-intensity, or an equivalent combination of endurance-oriented activity of both intensities. The activities should be performed in bouts of at least 10 minutes at a time, complemented by muscle strengthening physical activities on at least two days per week [6, 7, 8]. However, in the revised 2020 WHO physical activity guidelines, the requirement for sessions to last a minimum of 10 minutes was removed [9, 10].

Although no studies have yet explicitly investigated the extent to which the German population is aware of the WHO recommendations on physical activity, data from the first DEGS study conducted by the Robert Koch Institute (RKI) [2] indicate that 35 % of adults *pay close or very close attention to getting enough exercise.* Nevertheless, only 20 % actually meet the WHO’s physical activity recommendations. The discrepancy is even more pronounced among those aged 60 to 79: around 45 % state that they *pay close or very close attention to getting enough exercise*, yet only around 16 % reach the recommended level of activity [2].

These findings suggest that the general awareness of the positive effects of physical activity – even in older age – is widespread among the German population. At the same time, studies show that old age and older people are still often perceived in a deficit-oriented manner. They tend to be associated with inactivity, physical decline, and health deficits [11, 12]. Such negative views on aging imply that aging is characterized by an irreversible physical and mental decline that is hardly modifiable. When such generalized assumptions about the group of older adults – so-called *other-directed views on* aging – are internalized, they can develop into *self-directed views on aging* and, under certain conditions, trigger a process of self-fulfilling prophecy. If individuals internalize the belief early in life that aging is an inevitable process of biological decline, they may be less inclined to maintain physical activity throughout the life course – particularly in later life – which, as several studies have shown, is indeed associated with a decline in physical capacities [13, 14, 15].

**Key messages**

► Although age(ing) and older adults are often associated with health deficits, the prescriptive age norm of staying physically active in later life receives high levels of approval.
► The findings show that this norm is particularly endorsed by older adults compared to younger individuals.
► At the same time, significantly fewer older adults meet the level of health-enhancing physical activity recommended by the World Health Organization.
► The results indicate a ‘norm-behavior gap’ with expectations regarding physical activity levels in old age and actual activity levels diverging markedly, especially from around age 75 onwards.
► Possible explanations include internalized views on aging and a lack of age-appropriate opportunities for physical activity.

Overall, there is robust evidence that *ageism*, i.e., the unjustified and unequal evaluation and treatment of older adults based on their chronological age [16], is associated with a variety of physical and psychological complaints (see, for example, the meta-analysis by [17]). The consequences are particularly severe when individuals internalize ageism (so-called *internalized ageism*) and (consciously or unconsciously) come to view their own aging as deficient, hardly modifiable, and less valuable. While positive self-directed views on aging predict longer expectancy [18], several studies show the negative effects of negative views on aging on health-related behavior [19, 20].

In contrast, *prescriptive age norms* refer to normative expectations of how older adults *should behave*, in comparison to descriptive views on aging, which describe how older adults are perceived [21]. Prescriptive age norms outline an ideal image of what constitutes a ‘good old person’ and can shape both older adults, self-perceptions and their attitudes and behaviors in later life [22]. Research suggests that such prescriptive age norms tend to become increasingly internalized with age – older adults increasingly adopt them and align their behavior accordingly [21].

The prescriptive norm of staying active and fit in old age may be more strongly advocated today than it was in the past [21]. Qualitative studies from various industrialized countries indicate that older adults view maintaining physical activity as a key component of successful aging [23, 24, 25, 26]. Quantitative findings also show that older adults largely agree with the statement that adequate exercise can be expected for their age group. For example, in a survey of adults aged 55 years and older in Belgium, 85 % *strongly* or *very strongly* agreed with the statement ‘adults over 55 should exercise’ [27].

Many older adults have now internalized the health benefits of physical activity, translating this knowledge into an expectation to remain active themselves in later life and to expect the same from their peers. But are they, in fact, physically active in old age? Self-reported activity levels and expressed support for an active lifestyle among older adults do not always align with objectively measured levels of physical activity or adherence to exercise recommendations [25]. Studies have found that even when exercise is perceived as personally beneficial [24, 28] and culturally valued [23], subjective interpretations of what constitutes health-promoting activity can lead many older adults to fall short of meeting international exercise recommendations according to objective criteria [24, 26].

The aim of the present study was therefore to investigate, for Germany, the discrepancy between agreement with the prescriptive age norm of physical activity in old age and actual physical activity behavior. Specifically, the analysis explored whether the assumed ‘norm-behavior gap’ is more pronounced among older adults than among younger ones. This would be reflected in a growing divergence between increasing endorsement of the prescriptive age-related norm of physical activity and a simultaneous decline in actual activity levels with advancing age. To this end, data on the prescriptive norm of physical activity from the Age_ISM Germany dataset [12] were contrasted with data on physical activity behavior from the GEDA 2019/2020-EHIS dataset of the Robert Koch Institute [29]. Both datasets provide up-to-date, population-representative information for Germany, enabling a nuanced description of the prescriptive norm of remaining physically active in old age, as well as an analysis of actual compliance with the WHO physical activity recommendations across age groups.

## 2. Methods

### 2.1 Sample design and study implementation

The Age_ISM Germany study was a nationwide cross-sectional survey conducted by the MSB Medical School Berlin on behalf of the Federal Anti-Discrimination Agency (Antidiskriminierungsstelle des Bundes) [12]. The aim was to establish a sound empirical basis on which to analyze the perceptions, attitudes, and evaluations of the population living in Germany regarding older adults and the life stage of old age. To this end, a total of 2,000 individuals aged 16 years and older were surveyed by telephone across Germany in January 2022. A detailed description of the sampling and survey methodology is provided in Kessler and Warner [12].

The GEDA study has been conducted by the Robert Koch Institute on behalf of the Federal Ministry of Health (Bundesministerium für Gesundheit) since 2008 at intervals of several years as a nationwide cross-sectional telephone survey [30]. It provides current data on health status, health determinants, and the use of the health care services in Germany. For the present analysis, data from GEDA 2019/2020-EHIS, collected between April 2019 and September 2020 from 23,001 individuals aged 15 years and older, were used. A detailed description of the sampling and survey methodology is provided in Allen et al. [29].

### 2.2 Prescriptive age stereotypes on ‘physical activity’

The Age_ISM Germany study used adapted items from the inventory for recording prescriptive age stereotypes (expectations of older adults) [21], which include the expectation that older adults should, among other things, remain physically active. The statement used in the present study began with the phrase ‘Older people should normally…’ and ended with ‘…remain physically active.’ Agreement was measured using a 4-point response scale (1 = *strongly disagree*, 2 *= somewhat disagree*, 3 *= somewhat agree*, 4 *= strongly agree).*

### 2.3 Physical activity

Compliance with the WHO physical activity recommendations for adults (aged 18 – 64 years) was used as an indicator of the level of physical activity, with the same recommendations applying to adults aged 65 and older [10]. In GEDA 2019/2020-EHIS, data were collected using the German validated version of the European Health Interview Survey-Physical Activity Questionnaire (EHIS-PAQ) [31]. Information on leisure-time physical activity – including sports and fitness – was collected with respect to:

- Aerobic endurance activity: Physical activity leading to at least a slight increase in breathing or heart rate. For example: Nordic walking, ball sports, jogging, cycling, swimming, aerobics, rowing, or badminton. ‘How much time do you spend in total in a typical week doing sports, fitness, or physical activity in your free time?’
- Muscle strengthening: ‘On how many days in a typical week do you engage in physical activities specifically designed to build or strengthen your muscles? For example: strength training or strengthening exercises with weights, Thera-Band, your own body weight, squats, push-ups, or sit-ups.’

Compliance with the WHO recommendation in a typical week (yes, no) was defined as follows:

- Aerobic endurance activity: at least 150 minutes of moderate aerobic physical activity
- Muscle strengthening: at least 2 days/week covering all major muscle groups
- Compliance with both WHO recommendations (aerobic endurance activity and muscle strengthening)

### 2.4 Statistical methods

The Age_ISM Germany study data set was used for the statistical analysis of agreement with the prescriptive norm. A one-way analysis of covariance (ANCOVA) was conducted to examine the association between age and agreement with the statement that older people should normally remain physically active. The participants’ age group (16 – 24, 25 – 34, 35 – 44, 45 – 54, 55 – 64, 65 – 74, 75 – 84 years, 85 years and older) was included as a categorical independent variable. Gender (female, male, diverse) was included as a covariate in order to statistically control for potential gender-specific effects. The dependent variable was the degree of agreement with the statement (mean value). The aim of the analysis was to determine the effect of age group on agreement with the prescriptive norm, controlling for gender.

The statistical analysis of physical activity is based on data from the GEDA 2019/2020-EHIS study. To examine the relationship between age and self-reported physical activity, one-way analyses of covariance (ANCOVA) were also performed. The participants’ age group (18 – 24, 25 – 34, 35 – 44, 45 – 54, 55 – 64, 65 – 74, 75 – 84 years, 85 years and older) was included in the analysis as a categorical independent variable, while gender (female, male) was again considered as a covariate. The three dependent variables representing physical activity were: meeting the WHO recommendations for weekly i.) aerobic endurance activity, ii.) muscle strengthening, and iii.) meeting both aerobic endurance activity & muscle strengthening. The analyses aimed to determine the effect of age group on compliance with the individual and combined WHO physical activity recommendations, while controlling for gender.

Prior to conducting the analyses, all statistical assumptions for ANCOVA were checked. To identify significant differences between age groups more precisely, post-hoc comparisons with Bonferroni correction were applied to control for cumulative alpha errors in multiple pairwise comparisons. The significance level for all tests is p <. 05. Statistical analyses were performed using IBM SPSS Statistics version 28.0.1.1.

## 3. Results

In total, data from 986 women and 980 men (N = 1,966) aged 16 to 96 years from the Age_ISM Germany study were analyzed with respect to agreement with the prescriptive age norm. To assess physical activity, data from 11,968 women and 10,740 men (N = 22,708) aged 18 years and older from the GEDA 2019/2020-EHIS study were analyzed (participants under 18 were excluded, as different WHO physical activity recommendations apply to this age group).

**Figure 1:**
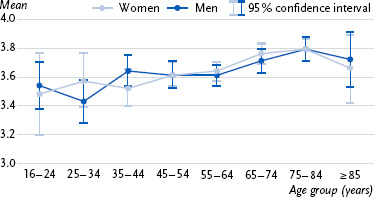
Average agreement (95 % confidence interval) with the statement ‘Older people should normally remain physically active’ by age group and gender (n = 986 women, n = 980 men). Agreement was measured on a four-point scale: 1 = strongly disagree, 2 = somewhat disagree, 3 = somewhat agree, 4 = strongly agree. Source: Age_ISM Germany, 2022

**Figure 2:**
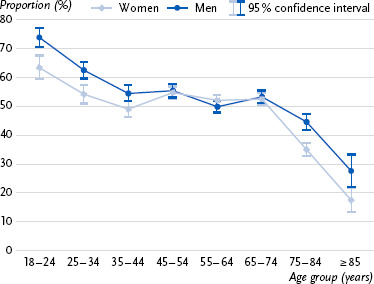
Proportion of individuals (%, 95 % confidence interval) in each age group who meet the WHO recommendation for weekly aerobic endurance activity (≥ 150 minutes, excluding walking) in Germany by gender (n = 11,825 women, n = 10,618 men). Source: GEDA 2019/2020-EHIS

**Figure 3:**
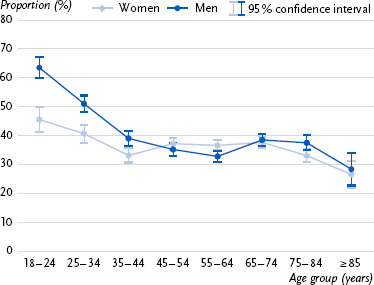
Proportion of individuals (%, 95 % confidence interval) in each age group who meet the WHO recommendation for weekly muscle strengthening (≥ 2 days a week) in Germany by gender (n = 11,922 women, n = 10,710 men). Source: GEDA 2019/2020-EHIS

**Figure 4:**
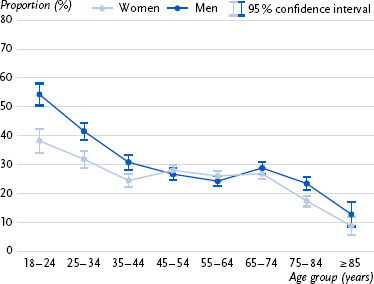
Proportion of individuals (%, 95 % confidence interval) in each age group who meet both WHO recommendations for weekly aerobic endurance activity (≥ 150 minutes, excluding walking) and muscle strengthening (≥ 2 units) in Germany by gender (n = 11,795 women, n = 10,594 men). Source: GEDA 2019/2020-EHIS

First, approval ratings for the prescriptive age norm across age groups are presented (Figure 1), followed by information on the three indicators for meeting the WHO recommendations for weekly physical activity (Figure 2, Figure 3, Figure 4). Detailed statistical parameters of the ANCOVA and post-hoc analyses, as well as mean values and confidence intervals, are provided in tabular form in the annex (see Annex Table 1, Annex Table 2, Annex Table 3, Annex Table 4).

### 3.1 Agreement with the statement ‘Older people should normally remain physically active’

The results indicate that agreement with the statement that older people should remain physically active was significantly associated with age (Figure 1). Older individuals tended to agree with this statement more than younger individuals. This difference was particularly pronounced when comparing the youngest and oldest age groups. Participants aged 16 to 34 showed lower agreement than those aged 65 and older. In the middle age groups, agreement was also lower in some cases compared with older respondents. Gender showed no significant correlation with agreement with the prescriptive norm.

### 3.2 Compliance with WHO recommendations for weekly aerobic endurance activity

Overall, a clear negative trend was observed, with compliance with the WHO recommendation for aerobic endurance activity decreasing markedly and, in some cases significantly, with increasing age (Figure 2). Younger individuals complied with the exercise recommendation significantly more often than older individuals. In particular, those aged 18- to 24 showed the highest compliance rate and differed significantly from all other age groups. While the compliance rate remained relatively stable among the 35 to 74 age groups, a significant decline was observed from the age of 75 onwards, which became significant in the 85+ age group. The results further indicate that, in addition to age, gender is also associated with compliance with the WHO recommendations for aerobic endurance activity. Women in the younger and oldest age groups were significantly less likely to meet the WHO exercise recommendations than men.

### 3.3 Compliance with WHO recommendation for weekly muscle strengthening

A clear negative age trend was also observed for compliance with the WHO recommendation for weekly muscle strengthening activity (≥2 days a week), with compliance decreasing significantly with increasing age. Younger age groups, particularly those aged 18 – 24, complied with the recommendations significantly more often than older individuals. In the middle to older age groups, compliance stabilized at a relatively low level of around 35 – 38 percent. The oldest age group, aged 85 and above, showed the lowest compliance rates and differed significantly from all other age groups. Furthermore, gender-specific differences were observed in the younger and oldest age groups, with men complying with the recommendations significantly more often than women.

### 3.4 Compliance with both WHO recommendations for weekly aerobic endurance activity & muscle strengthening

When combining the WHO recommendations for weekly aerobic endurance activity and muscle strengthening activity, both age and gender were associated with compliance. Younger individuals reported meeting the recommendations significantly more often, whereas a clear decline was observed with increasing age. The highest compliance rates were found in the 18 – 24 age group, while compliance was particularly low among those aged 85 and above. After an initial decline up to the 35 – 44 age group, compliance rates stabilized in middle age before declining again from age 75 and significantly from age of 85. The trend was confirmed for gender, with younger and older women being significantly less likely than men to report meeting both WHO physical activity recommendations in combination.

## 4. Discussion

A comparison of the two cross-sectional surveys reveals an age-dependent increase in agreement with the prescriptive norm of remaining physically active in old age, accompanied by a decline in actual compliance with the WHO recommendations for physical activity. Older individuals agreed more strongly than younger individuals with the statement that older people should remain physically active, indicating that prescriptive age norms are more firmly established in older age groups. At the same time, actual compliance with the WHO physical activity recommendations – both for aerobic endurance and muscle strengthening activities – decreased significantly with increasing age. The highest compliance rates were observed among young adults (18 – 24 years), whereas a significant decline was particularly evident in the age groups 75+ respectively 85+. In the middle age groups, compliance tended to plateau at a relatively low level, especially with regard to the recommendations for muscle strengthening.

Gender-specific differences also emerged: in several age groups, particularly the younger and oldest adults, women were significantly less likely than men to report meeting the WHO recommendations for endurance and muscle strengthening activities, as well as their combination.

Overall, the results indicate significant age- and gender-specific differences between the prescriptive norm and actual activity behavior. A clear norm-behavior gap is evident in older age, which appears to be more pronounced in women than in men. From the age of 75 onwards, regardless of gender, there is a marked discrepancy between expectations of physical activity behavior in old age and actual physical activity behavior.

From a public health perspective, the overall high level of agreement with the prescriptive norm of remaining physically active in old age can be interpreted positively. It can be assumed that knowledge about the health-promoting effects of physical activity is already relatively widespread in Germany, even among older age groups for whom this knowledge is particularly relevant. This is also reflected in a recent Belgian study, which found that respondents aged 55 and older who agreed more strongly with the prescriptive norm that older people should be active were significantly more motivated to engage in physical activity themselves and were more active in practice [27].

However, a strong prescriptive norm to remain active at any age can also convey expectations and demands. Socially and health-disadvantaged individuals exposed to such norms in old age may perceive them as excessive pressure or even a threat [33]. Future studies should place greater emphasis on the physical and mental health status of the respondents.

### 4.1 Limitations

The interpretation of the results is subject to several limitations that must be taken into account. First, the findings show relatively small effect sizes and are based on two different samples that differ in terms of survey timing and composition. Due to the cross-sectional study design of both data sets, these provide only snapshots and do not allow conclusions to be drawn about temporal developments or trends in older adults’ understanding of norms and physical activity behavior.

Second, the measurement of the prescriptive norm is limited: the question used in the Age_ISM Germany survey – ‘Older people should normally remain physically active’ – does not explicitly refer to the minimum level of health-promoting physical activity recommended by the WHO. Studies also show that older adults’ activity patterns are increasingly shifting from intensive sports to lighter forms, such as walking or household chores and gardening activities. These low-intensity activities were not considered in the present analysis. Therefore, it can be assumed that many participants have a broader understanding of the concept of ‘remaining physically active’ than is implied by the WHO’s physical activity recommendations.

Thirdly, the GEDA items provided only limited information for assessing actual activity levels in accordance with the WHO’s physical activity recommendations. As noted in previous studies [2, 31], the questions used allow only for an approximate estimate. For instance, higher-intensity activities that fall below 150 minutes of moderate activity (e.g. 75 minutes of intense activity) could not be taken into account. Finally, participants’ knowledge of the WHO physical activity recommendations was not explicitly assessed. Since awareness of specific recommendations for health-promoting physical activity is a key prerequisite for their implementation and individual motivation – particularly, but not exclusively, in older age – future studies should explicitly assess the corresponding level of knowledge among the German population.

Other potential influencing factors, such as education and health status, were not taken into account in the analyses. However, supplementary sensitivity analyses including education as a covariate showed that the main effects of age remained significant.

### 4.2 Possible causes for the norm-behavior gap in old age

The gap between normative behavior and actual behavior, which becomes more pronounced with age, can have multiple causes. At *the socio-structural level*, there is a lack of age-sensitive service structures that could motivate and enable older adults to engage in physical activity. For example, deficits remain in the German prevention landscape regarding services specifically designed for the heterogeneous group of older adults and tailored to their needs and demands for meaningful physical activity. In addition to traditional sports clubs, which now offer more health-oriented sports, fitness studios play a central role, as they increasingly present themselves as health-oriented service providers and are actively involved in promoting the image of ‘fit aging.’ However, only a few fitness studios actually take older adults’ needs into account – both in their advertising and the design of their service offerings. Aspects such as respectful communication, individual support, or a training environment that is not performance-oriented are often neglected or are available only in higher-priced studios. Analyses of gym websites and interviews with trainers and older users suggest that this can lead to the social exclusion of less privileged groups [34, 35]. Older adults are frequently not adequately assessed by trainers regarding their physical performance level or motivation to exercise, partly due to insufficient training and continuing education structures and the lack of widely implemented age-specific training concepts. This can result in over- or under-challenging older adults. Furthermore, not all sports and exercise programs in gyms, and in some cases sports clubs, are sufficiently barrier-free or low-threshold for older adults and can be very expensive. However, this is particularly relevant for older adults, who often cite unfavorable weather conditions, risky traffic, unattractive residential areas, and other challenging environment features (including poor lighting, missing or uneven sidewalks, structural decay) as barriers to physical activity [26, 36].

At the individual level, the decline in physical activity levels can be attributed to older adults withdrawing from at least moderately strenuous physical activity, as recorded in GEDA 2019/2020-EHIS. Safety concerns and fears of falling contribute to this withdrawal, as do existing challenges related to chronic health conditions, mobility limitations, and pain. All of these factors impair the subjective or actual ability to be physically active [26]. Negative views on aging also play a role, including conscious or unconscious assumptions that passivity is part of being old and that one inevitably becomes slower, frailer, and therefore less athletic with age. Another possible psychological barrier is the conscious or unconscious assumption that older adults do not belong in the gym or on sports teams. This phenomenon, referred to as *social awkwardness* [26], reflects another prescriptive age norm, according to which older adults should withdraw and avoid appearing youthful [12]. These factors may also contribute to the decline in sports club membership among older adults, with only 15.8 % of women and 27.8 % of men over 60 years of age being members of a sports club in Germany [37].

The findings of this study should also be interpreted in light of possible cohort effects, as reported in previous studies [3, 38]. The older cohorts in the data sets correspond to birth cohorts prior to 1955. It can therefore be assumed that their lower level of targeted health-promoting physical activity is also associated with the fact that these older generations were socialized under conditions in which health-promoting physical activity was less common and less expected. Prior experience with health-promoting physical activity (e.g. fitness, health, and rehabilitation sports) is, however, a key predictor of current activity levels [26, 39, 40]. Older adults thus tend to find themselves in a historical situation in which physical activity is socially expected, but few individual routines for health-promoting physical activity have been established.

### 4.3 Implications for programs to promote physical activity in older age

Based on the available research findings, a question for public health research is: How can the norm-behavior gap regarding physical activity in old age be reduced, and ideally, closed? Although the present data do not allow direct conclusions on how to improve the range of physical activity opportunities for older adults in Germany, the following section presents findings from the literature on how physical activity in later life can be made more appropriate and attractive. These considerations are based on the fact that interventions to promote physical activity among older population groups in non-clinical settings are often derived from studies of the general population. It can, however, be assumed that older adults require specifically tailored, age-sensitive measures and approaches, as their motivations, preferences, and physical capacities may differ substantially from those of younger adults.

### 4.4 Meaning orientation instead of a sense of duty

Behavior change techniques such as goal setting, planning, and self-monitoring are effective in adults under the age of 60 years [41], but have significantly less impact in adults aged 65 and above [42]. Nevertheless, many behavioral intervention approaches and exercise programs still rely on these self-regulatory strategies. For older adults, however, the focus is less on fulfilling exercise recommendations and more on enjoying the activity, social interaction, and noticeable, sometimes short-term effects such as improved mood through physical activity [26, 36, 43, 44]. A systematic review by Reich et al. (2020), which summarized qualitative data from 13 countries, underscores this point: Older adults (aged 50 – 98) mention social integration and a positive attitude significantly more often than physical health or independence when describing ‘successful aging’ [45]. Furthermore, inactive older adults, in particular, report feeling intimidated by fitness facilities or concerned about hindering others in (sports) groups, highlighting that meaning and enjoyment are central to their engagement in physical activity [26, 28].

The communication strategy should therefore not be ‘Sport and exercise is healthy,’ but rather ‘Exercise is fun, brings people together – and is also good for the body.’ The focus should be less on indication-specific health sports (e.g. fall prevention or heart health) and more on how exercise helps individuals cope with daily activities independently and maintain social participation. Ideally, promoting physical activity in older age should be designed to be relevant to everyday life and participation-oriented, and should be based on individual motives rather than performance standards [26].

### 4.5 Integration into everyday life instead of an additional task

‘Some physical activity is better than none’ – With the revision of its 2020 recommendations, the WHO emphasizes that ‘any movement is better than none.’ The previous minimum duration of 10 minutes for an endurance session has been removed, and definitions of strength and balance exercises have been adjusted. For the first time, the guidelines also encourage reducing sitting and inactivity, highlighting that more movement brings additional health benefits. Using these updated WHO guidelines to integrate physical activity into meaningful everyday tasks and for personal enjoyment may better meet the needs of older adults than if physical activity is perceived as an additional obligation. A meta-synthesis of ten qualitative studies [36] shows that older adults often view physical activity as a by-product of other activities rather than as a goal-oriented activity (e.g. in the sense of systematic training). This may lead to a reduced willingness to engage in physical activity when exercise competes with social roles (e.g. household, family, volunteer work) [26]. Accordingly, the promotion of exercise and physical activity should be easily integrated into daily life without being perceived as another ‘to-do’.

### 4.6 Consider digitalization and online exercise programs

Free online programs in Germany such as ‘Gesund & aktiv älter werden’ (Staying healthy and active as you age) [46] or the ‘Fit im Nordwesten’ (Fit in the Northwest) toolbox [47] are particularly suitable for older adults with limited mobility or for individuals in regions with poor infrastructure who cannot or do not wish to use nearby exercise programs. However, barriers to access can prevent participation in such digital exercise programs, such as a lack of internet connection, absence of internet-enabled devices (smartphone, tablet, computer, smart TV, etc.), or limited digital skills. Purchase and maintenance costs for devices and internet services may also act as obstacles. Targeted measures are therefore necessary to improve participation in digital exercise programs, such as the nationwide expansion of internet infrastructure and financial support for older adults with limited resources. Commercial providers in Germany now offer low-tech devices with internet access specifically for older target groups, which could facilitate access to online exercise programs in the future. In this context, *exergaming* also appears to be an innovative approach to promoting exercise. Exergaming combines physical training (e.g. strength and endurance) and cognitive training (e.g. dual tasking, memory training) with playful elements. It can be done on a computer, tablet, game console, or using VR glasses, and can be adapted to different age groups [48], while still achieving moderate physical exertion [49]. Digital exercise programs thus have the potential to contribute to meeting the WHO’s physical activity recommendations.

### 4.7 Conclusion

The contradictory trend – high agreement with the statement that older people should remain physically active and the empirical observation that fewer individuals meet the WHO’s physical activity recommendations as they age – points to individual and structural barriers. Promoting physical activity in old age often appears to be conceived within a context that requires high physical, economic, and cultural resources [50]. This creates the risk that the prescriptive age norm established less as an inclusive health goal and more as a selection criterion that reinforces social inequalities in access to health-promoting services. The expectation of activity in later life is thus not only normatively exaggerated, but also carries the potential to become a new form of social pressure, particularly excluding those who cannot or do not wish to meet the high demands of ‘active and successful aging.’ There is therefore a need for low-threshold, widely accessible exercise programs that are adapted to the needs and preferences of older target groups.

## Figures and Tables

**Annex Table 1a:** ANCOVA results and significant post-hoc comparisons: Agreement with “Older people should normally remain physically active” (n = 986 women, n = 980 men). Source: Age_ISM Germany, 2022

ANCOVA	Group comparison	*F*(df)	*p*-value	*η^2^_p_*
Main effect	Age groups	*F*(7, 1957) = 6.72	<.001	.022
Covariates	Gender	*F*(1, 1962) = 0.19	.662	**–**
**Post-hoc comparisons**	**Group comparison**	***M*_DIFF_**	***p*-value**	**(95 % CI)**
	16 – 24 vs. 65 – 74 years	-0.22	.034	(-0.43 – -0.01)
	16 – 24 vs. 75 – 84 years	- 0.27	.005	(-0.49 – -0.05)
	25 – 34 vs. 65 – 74 years	- 0.25	<.001	(-0.42 – -0.08)
	25 – 34 vs. 75 – 84 years	- 0.30	<.001	(-0.49 – -0.12)
	45 – 54 vs. 75 – 84 years	- 0.17	.023	(-0.32 – -0.02)
	55 – 64 vs. 75 – 84 years	- 0.16	.008	(-0.30 – -0.02)

df = Degrees of freedom, *p*<.05 = significant, *η^2^_p_ =* partial eta square, *M*_DIFF_ = mean difference, CI = confidence interval

**Annex Table 1b:** Agreement with “Older people should normally remain physically active” by gender and age group (n = 986 women, n = 980 men) Response options: 1 = strongly disagree, 2 = somewhat disagree, 3 = somewhat agree, 4 = strongly agree. Source: Age_ISM Germany, 2022

	Mean	(95 % CI)
**Total (n = 1,968)^1^**	**3.64**	**(3.62 – 3.67)**
Women (n = 986)	3.66	(3.62 – 3.69)
Men (n = 980)	3.63	(3.60 – 3.67)
**Age group**		
**Total**		
16 – 24 years (n = 85)	3.52	(3.38 – 3.65)
25 – 34 years (n = 151)	3.48	(3.37 – 3.60)
35 – 44 years (n = 204)	3.60	(3.51 – 3.67)
45 – 54 years (n = 334)	3.61	(3.55 – 3.68)
55 – 64 years (n = 552)	3.63	(3.58 – 3.67)
65 – 74 years (n = 364)	3.74	(3.69 – 3.79)
75 – 84 years (n = 224)	3.79	(3.73 – 3.84)
≥ 85 years (n = 54)	3.69	(3.54 – 3.83)
**Women**		
16 – 24 years (n = 27)	3.48	(3.20 – 3.76)
25 – 34 years (n = 54)	3.57	(3.39 – 3.76)
35 – 44 years (n = 86)	3.52	(3.40 – 3.65)
45 – 54 years (n = 181)	3.61	(3.53 – 3.70)
55 – 64 years (n = 290)	3.64	(3.57 – 3.70)
65 – 74 years (n = 198)	3.76	(3.69 – 3.83)
75 – 84 years (n = 121)	3.79	(3.71 – 3.86)
≥ 85 years (n = 29)	3.66	(3.42 – 3.89)
**Men**		
16 – 24 years (n = 57)	3.54	(3.38 – 3.70)
25 – 34 years (n = 97)	3.43	(3.28 – 3.58)
35 – 44 years (n = 118)	3.64	(3.54 – 3.75)
45 – 54 years (n = 152)	3.61	(3.52 – 3.71)
55 – 64 years (n = 262)	3.61	(3.54 – 3.68)
65 – 74 years (n = 166)	3.71	(3.63 – 3.79)
75 – 84 years (n = 103)	3.79	(3.71 – 3.87)
≥ 85 years (n = 25)	3.72	(3.53 – 3.91)

CI = confidence interval

^1^Only two people specified the category “Other,” so no group-based evaluation was performed for this category. Both individuals are included in the evaluation across all genders.

**Annex Table 2a:** ANCOVA results and significant post-hoc comparisons: Compliance with the WHO recommendation for ≥ 150 minutes of weekly aerobic endurance activity (excluding walking) (n = 11,825 women, n = 10,618 men). Source: GEDA 2019/2020-EHIS

ANCOVA	Group comparison	*F*(df)	*p*-value	*η^2^_p_*
Main effect	Age groups	*F*(7, 22434) = 84.94	<.001	.026
Covariates	Gender	*F*(1, 22434) = 23.08	<.001	.001
**Post-hoc comparisons**	**Group comparison**	***M*_DIFF_**	***p*-value**	**(95 % CI)**
	18 – 24 vs. all other age groups	**>**	<.001	–
	25 – 34 vs. all older age groups, except 45 – 54 years	**>**	<.001	–
	35 – 44 vs. 75-84 years	12.48	<.001	(8.33 – 16.63)
	35 – 44 vs. ≥ 85 years	29.82	<.001	(22.71 – 36.93)
	45 – 54 vs. 75-84 years	15.90	<.001	(12.11 – 19.69)
	45 – 54 vs. ≥ 85 years	33.24	<.001	(26.33 – 40.15)
	55 – 64 vs. 75 – 84 years	11.49	<.001	(7.51 – 15.47)
	55 – 64 vs. ≥ 85 years	29.23	<.001	(22.44 – 36.03)
	65 – 74 vs. 75 – 84 years	13.70	<.001	(9.99 – 17.41)
	65 – 74 vs. ≥ 85 years	31.05	<.001	(24.18 – 37.91)
	75 – 84 vs. ≥ 85 years	17.34	<.001	(10.30 – 24.39)

df = Degrees of freedom, *p*<.05 = significant, *n^2^_p_ =* partial eta square, M_DIFF_ = mean difference, CI = confidence interval

**Annex Table 2b:** Compliance with the WHO recommendation for ≥ 150 minutes of weekly aerobic endurance activity (excluding walking) by gender and age group, percentage and 95 % CI (n = 11,825 women, n = 10,618 men). Source: GEDA 2019/2020-EHIS

	%	(95 % CI)
**Total (n = 22,632)**	**51.54**	**(50.89 – 52.20)**
Women (n = 11,922)	49.62	(48.71 – 50.52)
Men (n = 10,710)	53.69	(52.74 – 54.64)
**Age group**		
**Total**		
18 – 24 years (n = 1,172)	69.30	(66.65 – 71.94)
25 – 34 years (n = 2,040)	58.84	(56.71 – 60.99)
35 – 44 years (n = 2,643)	51.68	(49.77 – 53.60)
45 – 54 years (n = 3,844)	55.10	(53.52 – 56.68)
55 – 64 years (n = 5,115)	51.08	(49.71 – 52.47)
65 – 74 years (n = 4,290)	52.90	(51.40 – 54.41)
75 – 84 years (n = 2,949)	39.20	(37.43 – 40.98)
≥ 85 years (n = 579)	21.85	(18.46 – 25.25)
**Women**		
16 – 24 years (n = 516)	63.43	(59.25 – 67.60)
25 – 34 years (n = 912)	54.24	(50.99 – 57.48)
35 – 44 years (n = 1,367)	49.08	(46.43 – 51.74)
45 – 54 years (n = 2,073)	54.79	(52.64 – 56.95)
55 – 64 years (n = 2,753)	52.11	(50.23 – 53.98)
65 – 74 years (n = 2,295)	52.53	(50.48 – 54.59)
75 – 84 years (n = 1,674)	35.05	(32.75 – 37.35)
≥ 85 years (n = 332)	17.58	(13.45 – 21.70)
**Age group**		
**Men**		
16 – 24 years (n = 656)	73.89	(70.53 – 77.27)
25 – 34 years (n = 1,128)	62.56	(59.73 – 65.39)
35 – 44 years (n = 1,276)	54.48	(51.73 – 57.23)
45 – 54 years (n = 1,771)	55.45	(53.13 – 57.78)
55 – 64 years (n = 2,362)	49.89	(47.87 – 51.92)
65 – 74 years (n = 1,995)	53.32	(51.12 – 55.52)
75 – 84 years (n = 1,275)	44.64	(41.89 – 47.39)
≥ 85 years (n = 247)	27.69	(22.01 – 33.36)

CI = confidence interval

**Annex Table 3a:** ANCOVA results and significant post-hoc comparisons: Compliance with the WHO recommendation for weekly muscle strengthening (≥ 2 units) (n = 11,922 women, n = 10,710 men). Source: GEDA 2019/2020-EHIS

ANCOVA	Group comparison	*F*(df)	*p*-value	*η^2^_p_*
Main effect	Age groups	*F*(7, 22623) = 40.37	<.001	.012
Covariates	Gender	*F*(1, 22623) = 10. 97	<.001	.000
**Post-hoc comparisons**	**Group comparison**	***M*_DIFF_**	***p*-value**	**(95 % CI)**
	18 – 24 vs. 25 – 34 years	9.21	<.001	(3.69 – 14.73)
	18 – 24 vs. all other age groups	**>**	<.001	–
	34 – 44 vs. all other age groups	**>**	<.001	–
	55 – 64 vs. 65 – 74 years	- 3.22	.035	(-6.34 – -0.11)
	75 – 84 vs. ≥ 85 years	7.67	.013	(0.83 – 14.51)
	≥ 85 years vs. all other age groups	**<**	<.05	–

df = Degrees of freedom, *p*<.05 = significant, *η^2^_p_ =* partial eta square, *M*_DIFF_ = mean difference, CI = confidence interval

**Annex Table 3b:** Compliance with the WHO recommendation for weekly muscle strengthening (≥ 2 days a week) by gender and age group, percentage and 95 % CI (n = 11,922 women, n = 10,710 men). Source: GEDA 2019/2020-EHIS

	%	(95 % CI)
**Total (n = 22,389)**	**37.76**	**(37.13 – 38.39)**
Women (n = 11,795)	36.42	(35.56 – 37.28)
Men (n = 10,594)	39.25	(38.33 – 40.18)
**Age group**		
**Total**		
18 – 24 years (n = 1,172)	55.63	(52.79 – 58.48)
25 – 34 years (n = 2,040)	46.42	(44.26 – 48.59)
35 – 44 years (n = 2,643)	36.00	(34.16 – 3782)
45 – 54 years (n = 3,844)	36.26	(34.75 – 37.79)
55 – 64 years (n = 5,115)	34.82	(33.33 – 36.13)
65 – 74 years (n = 4,290)	38.04	(36.59 – 39.50)
75 – 84 years (n = 2,949)	34.96	(33.24 – 36.69)
≥ 85 years (n = 579)	27.29	(23.65 – 29.28)
**Women**		
16 – 24 years (n = 516)	45.54	(41.23 – 49.85)
25 – 34 years (n = 912)	40.68	(37.49 – 43.87)
35 – 44 years (n = 1,367)	33.14	(30.64 – 35.64)
45 – 54 years (n = 2,073)	37.19	(35.11 – 39.27)
55 – 64 years (n = 2,753)	36.58	(34.78 – 38.38)
65 – 74 years (n = 2,295)	37.65	(35.66 – 39.63)
75 – 84 years (n = 1,674)	33.03	(30.78 – 35.29)
≥ 85 years (n = 332)	26.51	(21.73 – 31.28)
**Men**		
16 – 24 years (n = 656)	63.57	(59.87 – 67.26)
25 – 34 years (n = 1,128)	51.06	(48.14 – 53.99)
35 – 44 years (n = 1,276)	39.03	(36.35 – 41.71)
45 – 54 years (n = 1,771)	35.18	(32.95 – 37.40)
55 – 64 years (n = 2,362)	32.77	(30.87 – 34.66)
65 – 74 years (n = 1,995)	38.50	(36.36 – 40.63)
75 – 84 years (n = 1,275)	37.49	(34.83 – 40.15)
≥ 85 years (n = 247)	28.34	(22.68 – 34.00)

CI = confidence interval

**Annex Table 4a:** ANCOVA results and significant post-hoc comparisons: Compliance with the WHO recommendations for weekly aerobic endurance activity and muscle strengthening (n = 11,795 women, n = 10,594 men). Source: GEDA 2019/2020-EHIS

ANCOVA	Group comparison	F(df)	p-value	*η^2^_p_*
Main effect	Age groups	*F*(7, 22380) = 70.62	<.001	.022
Covariates	Gender	*F*(1, 22380) = 27.03	<.001	.001
**Post-hoc comparisons**	**Group comparison**	***M*_DIFF_**	***p*-value**	**(95 % CI)**
	18 – 24 vs. ≥ 85 years	36.71	<.001	(29.66 – 43.76)
	18 – 24 vs. all older age groups	**>**	<.001	**-**
	25 – 34 vs. all older age groups	**>**	<.001	**-**
	75 – 84 vs. ≥ 85 years	-9.51	<.001	(-15.82 – – 3.19)
	75 – 84 and ≥ 85 vs. all younger age groups	**<**	<.05	**-**

df = Degrees of freedom, *p*<.05 = significant, *n^2^_p_ =* partial eta square, *M*_DIFF_ = mean difference, CI = confidence interval

**Annex Table 4b:** Compliance with the WHO recommendations for weekly aerobic endurance activity and muscle strengthening by gender and age group, percentage and 95 % CI (n = 11,795 women, n = 10,594 men) Source: GEDA 2019/2020-EHIS

	%	(95 % CI)
**Total (n = 22,389)**	**27.54**	**(26.95 – 29.13)**
Women (n = 11,795)	25.65	(24.87 – 26.44)
Men (n = 10,594)	29.63	(28.76 – 30.50)
**Age group**		
**Total**		
18 – 24 years (n = 1,167)	47.22	(44.35 – 50.08)
25 – 34 years (n = 2,031)	37.22	(35.12 – 39.33)
35 – 44 years (n = 2,620)	27.52	(25.81 – 29.23)
45 – 54 years (n = 3,812)	27.41	(26.00 – 28.83)
55 – 64 years (n = 5,060)	25.24	(24.04 – 26.43)
65 – 74 years (n = 4,230)	27.78	(26.43 – 29.13)
75 – 84 years (n = 2,898)	20.01	(18.56 – 21.47)
≥ 85 years (n = 571)	10.51	(7.99 – 13.03)
**Women**		
16 – 24 years (n = 513)	38.21	(33.99 – 42.43)
25 – 34 years (n = 907)	31.86	(28.83 – 34.90)
35 – 44 years (n = 1,361)	24.47	(22.18 – 26.75)
45 – 54 years (n = 2,052)	28.02	(26.08 – 29.97)
55 – 64 years (n = 2,725)	26.06	(24.41 – 27.70)
65 – 74 years (n = 2,263)	26.87	(25.04 – 28.69)
75 – 84 years (n = 1,645)	17.39	(15.55 – 19.22)
≥ 85 years (n = 329)	8.81	(5.74 – 11.89)
**Men**		
16 – 24 years (n = 654)	54.28	(50.45 – 58.11)
25 – 34 years (n = 1,124)	41.55	(38.66 – 44.43)
35 – 44 years (n = 1,259)	30.82	(28.26 – 33.37)
45 – 54 years (n = 1,760)	26.70	(24.64 – 28.77)
55 – 64 years (n = 2,335)	24.29	(22.54 – 26.02)
65 – 74 years (n = 1,967)	28.83	(26.82 – 30.83)
75 – 84 years (n = 1,253)	23.46	(21.11 – 25.81)
≥ 85 years (n = 242)	12.81	(8.57 – 17.05)

CI = confidence interval
